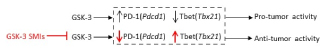# Commentary: Small Molecule Inhibition of PD-1 Transcription is an Effective Alternative to Antibody Blockade in Cancer Therapy

**DOI:** 10.29245/2578-3009/2019/1.1167

**Published:** 2019-05-07

**Authors:** Alison Taylor, Christopher E. Rudd

**Affiliations:** 1 Leeds Institute of Medical Research, University of Leeds, School of Medicine, Wellcome Trust Brenner Building, St James’s University Hospital, LEEDS LS9 7TF, UK; 2 Division of Immunology-Oncology Research Center, Maisonneuve-Rosemont Hospital, Montreal, Quebec H1T 2M4, Canada; 3 Département de Medicine, Université de Montréal, Montreal, Quebec H3C 3J7, Canada

The past few years has witnessed exciting progress in the application of “immune check-point inhibitors” (ICI) in the treatment of various human cancers^1^–^3^. This involves the use of antibody blockade with monoclonal antibodies (mAbs) that block receptor binding to their natural ligands. Programmed cell death-1 (PD-1) recognises PD ligand (PDL)-1 and PDL-2 on presenting cells and this sends signals that inhibit T-cell activation and effector cytotoxic responses. Through these mechanisms, PD-1 inhibits the immune system and can prevent autoimmune diseases 4. Tumor cells expressing PDL-1/PD-L2 can use this mechanism to evade immune surveillance, allowing disease progression. A therapeutic approach involves administration of mAbs that block the engagement of checkpoint molecules with their ligand. In the case of anti-PD-1, these mAbs block the binding of PD-1 on the T-cell with PDL-1/PDL-2 on the tumor cell, preventing recognition and allowing activation of the T-cell to provide an immune response against the tumor cell. Blockade also reverses T-cell exhaustion and restores T-cell functionality 5, 6. Furthermore, PD-1 expression on tumor-infiltrating CD8^+^ T-cells correlates with impaired function, while PD-L1 expression on tumors facilitates escape^4^.

One of the first established immunotherapeutic approaches involved the use of Ipilimumab against CTLA-4^7^, ^8^. It was the prototypical immunomodulatory antibody first approved by the FDA in 2011 for advanced melanoma based on its survival benefit. This was followed by the highly successful blockade of PD-1 (i.e. Nivolumab and Pembrolizumab), or its ligand (PD-L1) (i.e. Atezolizumab), either alone^7^, or in combination with anti-CTLA-4^8^. In certain cases, the use of PD-1 mAbs superseded CTLA-4 mAbs, due to their increased response rates^9^, ^10^ and the combination of both therapies gave rise to even superior response rates^10^, ^11^. However, this success correlated with increased toxic side effects. A substantial proportion of patients receiving ICI develop immune-related adverse events (irAEs) including colitis, endocrinopathies, hepatitis, pneumonitis, cardiotoxicity, nephritis, skin eruptions and vitiligo^12^–^20^. These events have been reported at 20-28%, 17-21% and 45-59% for the use of anti-CTLA-4, anti-PD1 or combination therapy, respectively^9^–^11^. These drugs are currently being used in the treatment of various cancers including Melanoma, renal cell carcinoma, colorectal cancer and Hodgkin lymphoma^21^–^24^ as well as the viral infection HCV^25^. Immune-modulating agents, such as corticosteroid, infliximab, and mycophenolic acid are being used to manage irAEs^26^ where possible but in some cases, treatment is discontinued.

Although some success has been seen, the majority of patients are still not cured, some develop resistance and those with immune-resistant cancers such as colon and ovarian are poorly responsive. This poor prognosis highlights a need to improve current or identify alternative clinical interventions.

As PD-1 plays a prominent role in immunotherapy, one approach for enhanced anti-tumor immunity would be to inhibit pathways that control the expression of inhibitory co-receptors such as PD-1. We are the first to show that the serine/threonine kinase glycogen synthase kinase 3 (GSK3) is a central regulator of PD-1 expression in CD8^+^ T cells.

There are two isoforms of GSK-3, GSK-3α and GSK-3β, which are encoded by separate genes, with highly homologous kinase domains (98% identity) but divergent N- and C-terminal regions^27^, ^28^. Both forms have been implicated in processes ranging from glycogen metabolism to gene transcription, apoptosis and microtubule stability. The notable aspect of GSK-3 is that it is constitutively active in resting T-cells and is inhibited by receptor induced activation signals^29^.

In this regard, we have shown that small molecule inhibitors (SMIs) of GSK-3 are effective in promoting viral clearance^30^ and our current work^31^ shows that GSK-3 SMI inhibition of *Pdcd1* (PD-1) transcription with a small molecule inhibitor (i.e. SB415286) is as effective as anti-PD-1 and PDL-1 blocking antibodies in the control of B16 and EL-4 tumor growth. Similar effects were observed using other inhibitors including SB216763 and CHIR99021 as well as the peptide inhibitor L803-mts. The exception was the inhibitor TWS119 which has been reported to retain cells in a less mature state^32^,^33^, by promoting the expression of TCF-1, blocking CD8+ T-cell differentiation, and inhibiting IFN-γ production^32^,^34^. Whereas other SMIs including SB415286 have been seen to promote differentiation and IFNγ production^30^,^35^–^36^. This difference between SMIs in their action on T-cell function underlines the need for defining the pathways of GSK-3 in T-cell signaling.

Our current work demonstrated that SB415286 significantly reduced B16 pulmonary metastasis. This anti-tumor effect of SB415286 was comparable to that using anti-PD-1 blocking antibody and combination of the two had no additional effect indicating an overlap in the two pathways. Further to this GSK-3 deficient T-cells from conditional knockout mice significantly reduced tumor progression confirming a direct role for GSK-3 in modulating anti-tumor activity in CD8^+^ T-cells. Our findings showed that GSK-3 inhibition operated primarily via a reduction in PD-1 expression on CD8^+^ T-cells. Inactivation of GSK-3 either through SMIs or by using GSK-*3α/β siRNA* led to a reduction in PD-1 expression and in both cases reduced B16 pulmonary metastasis to a similar extent as seen in *Pdcd1-/- mice.* In each model, GSK-3 SMIs inhibited *Pdcd1* transcription and PD-1 expression on tumor infiltrating T-cells (TILs), while increasing *Tbx21* (T-bet) transcription^30^ and the presence of CD8^+^ TILs expressing CD107a (LAMP1), granzyme B (GZMB) and IFNγ 1^31^. Other transcription factors such as Eomes (Eomesodermin) or the high mobility group (HMG) box Transcription factor 7 (Tcf7) were not affected.

Mechanistically, GSK-3 inactivation in T-cells with down-regulated T-bet had no effect on PD-1 expression indicating that GSK-3 operates upstream and is dependent on T-bet which in turn inhibits PD-1 expression. Further, down-regulation of T-bet increased PD-1 transcription indicating that T-bet suppresses the transcription of PD-1, in accord with results from the Wherry group^37^. High levels of T-bet expression could sustain exhausted CD8^+^ T-cells and repressed the expression of inhibitory receptors during chronic viral infection. Persistent antigenic stimulation caused downregulation of T-bet, which resulted in more severe exhaustion of CD8^+^ T-cells^37^.

Overall, in our model, active GSK-3 present in resting T-cells acts to supress *Tbx21* transcription. Upon T-cell activation GSK-3 becomes partially inactivated leading to partial T-bet expression and PD-1 suppression. The use of GSK-3 SMIs can fully repress GSK-3 leading to increased T-bet expression and complete inhibition of PD-1 expression.

The development of small molecules that modulate co-receptors or their signaling pathways to enhance anti-tumor activity would be a major advance in therapy. There are potential advantages and disadvantages to the use of GSK-3 inhibitors versus anti-PD-1 antibody therapies. Small molecules have the advantage of lower cost, dosing and potential oral administration. Further, anti-PD-1 is associated with a high cost as well as adverse effects such as fatigue, rash and possible autoimmune complications such as colitis. Although we cannot exclude these effects with GSK-3 SMIs, to date, we have seen no evidence of autoimmunity in the GSK-*3α/β-/-* mice over 2 years. The disadvantage of GSK-3 inactivation is the potential of an effect on the function of other host cells or the tumor itself. However, lithium chloride, another GSK-3 inhibitor, has been used for decades for the treatment of bipolar disease. Importantly, we showed that GSK-3 inhibition could affect PD-1 expression on both murine and human T-cells. The dose (200ug per 20g mouse) used is roughly comparable to the dose of another inhibitor Tideglusib which was used in a phase 2 oral study (800mg in a 80kg patient) to treat progressive supranuclear palsy^38^. Further, we showed that a single dose of SB415286 down-regulated PD-1 for 10-14 days. Although, we failed to see any effect of SB415286 directly on the growth of B16 melanoma cells in the absence of an immune response, GSK-3 inhibition has been reported to directly inhibit the growth of multiple myeloma, neuroblastoma, hepatoma and prostate tumors^38^–^43^. It is therefore possible that GSK-3 SMIs might have an added advantage by directly inhibiting the growth of some tumors in addition to enhancing the immune response. Despite these possibilities, the major effect of GSK-3 SMIs in our studies involved the amplification of the immune system as shown by the effects on *ex vivo* T-cells and adoptive transfer experiments as well as by the elimination of tumors in mice where T-cells have conditionally deleted *GSK-3α/β*. Certain tumors can impair proximal TCR signaling events as a form of immune avoidance^44^,^45^. The inhibition of GSK-3 could potentially circumvent this impairment given that GSK-3 operates down-stream of proximal signal mediators such as p56^lck^. Overall, our findings identify a potential alternate approach using small molecule inhibition of PD-1 expression in cancer immunotherapy. Further work is needed to uncover the full range of down-stream effects that may be regulated by GSK-3 regulation in anti-tumor immunity.